# Solid-state bonding behavior between surface-nanostructured Cu and Au: a molecular dynamics simulation

**DOI:** 10.1038/s41598-022-17119-w

**Published:** 2022-07-26

**Authors:** Hiroaki Tatsumi, C. R. Kao, Hiroshi Nishikawa

**Affiliations:** 1 Joining and Welding Research Institute, Osaka University, 11-1 Mihogaoka, Ibaraki, Osaka 567-0047 Japan; 2 Department of Materials Science and Engineering, National Taiwan University, No. 1, Sec. 4, Roosevelt Road, Taipei, 10617 Taiwan

**Keywords:** Surfaces, interfaces and thin films, Metals and alloys, Atomistic models

## Abstract

In recent years, solid-state bonding has attracted attention for various electronic packaging applications as an alternative to conventional solders. Surface-nanostructured materials enable solid-state bonding without complex surface modifications and operate at a low bonding temperature and pressure. Therefore, in this study, molecular dynamics simulations were conducted to investigate the solid-state bonding behavior between surface-nanostructured Cu and Au, with a focus on diffusion phenomena. A periodic ligament-cavity nanostructured Cu (NS-Cu) model was prepared at the bonding interface between Cu and Au slabs. The simulation results indicated that the larger the specific surface area of NS-Cu, the faster the densification at the bonding interface. Atomic displacement analysis showed that rapid densification occurred via the displacement of Cu and Au atoms in the vicinity of NS-Cu. The preferential diffusion of atoms along NS-Cu cavities contributed to this phenomenon. At this stage of densification, the diffusion coefficients were higher than the surface diffusion coefficients estimated based on literature, which indicates that this behavior is specific to surface-nanostructured materials. The highly disordered atomic arrangement at the bonding interface enabled significant atomic diffusion. Therefore, this study confirmed that the use of surface-nanostructured materials would contribute to a promising bonding technology for application in electronics.

## Introduction

In recent years, an increasing demand for electronic devices with a high degree of integration and current density has emerged. Sn-based solder joints, which are widely used to bond electronic devices, are approaching their theoretical limits. For example, in the latest three-dimensional integrated circuits (3D-ICs), the input/output (I/O) pitch is expected to decrease to 1 µm^1^. Conventional Sn-based solder bumps, whose minimum pitch is typically 20 µm^2^, possess drawbacks, such as potential failure caused by electrical-short circuits owing to contact between neighboring microbumps during bonding. As the volume of solder bumps decreases, the brittle intermetallic compounds (IMCs) and Kirkendahl voids formed at the joint interface demonstrate an increasing effect on joint characteristics. IMCs and Kirkendall voids restrict the reliability of solder joints^3^. Moreover, solder joints limit the applicable current density owing to electromigration^4^. Therefore, solid-state bonding technologies have been developed for application in high-end 3D-ICs^5–8^. Among these, solid-state Cu bonding has attracted particular attention. Cu solid-state bonding is achieved via room-temperature bonding^9^ or room-temperature bonding combined with post-annealing^5,6^. This bonding technology requires activated surfaces that are morphologically flat for improved bonding contact, which is provided through chemical mechanical polishing (CMP) and complex surface activation processes. However, this bonding approach is expensive and time-consuming. The thermal compression process is another route to realizing Cu solid-state bonding^7,8,10^. However, the high bonding pressure used in this type of processing limits possible applications.

Power devices are another significant facet in solid-state bonding. The die-attach process is a major challenge in high-temperature power devices^11^. For a typical Sn-based solder, the maximum durable temperature is generally limited to temperatures below 448 K because of its poor thermal-fatigue tolerance at high temperatures^12^. However, the operating temperature of power devices tends to increase with increasing current density, with an expected elevation in the operating temperature up to 473 K or higher. Unlike high-end 3D-ICs, power devices require materials with large-area bonding capability (several square millimeters) and thermal-strain tolerance, which is induced by a coefficient of thermal expansion (CTE) mismatch and temperature variations during operation. Therefore, instead of soldering, we envision another solid-state bonding approach for power die-attach applications: a bonding process using metal particles. This bonding process utilizes the phenomenon of the sintering of nano- or micro-sized metal particles^13–15^. Nano- or micro-sized particles of Ag or Cu with a highly reactive surface are typically used, which enables bonding at relatively low temperatures of approximately 573 K^16,17^. This method affords excellent joint properties, such as a good thermal-fatigue performance and low thermal resistance in power devices^18^. However, particle-based bonding materials, which are often used in the form of a paste mixed with organic matter, possess drawbacks, such as high costs, complex bonding processes, and void formation caused by the organic components in the paste.

To overcome these issues, a novel solid-state bonding method that utilizes nanoporous materials, such as Au nanoporous bumps^19^, Ag nanoporous sheets^20^, Cu nanoporous sheets^21^, and patterned Cu nanoporous caps^22^, has recently been investigated. These nanoporous materials are fabricated via the electrochemical dealloying of a precursor composed of noble and base metals; for example, Au–Ag^19^, Ag–Al^20^, Cu–Mg^21^, and Cu–Zn^22^. Nanoporous materials have network-like structures connected by nanometer-sized ligaments and nodes. Typically, nanoporous materials are extremely surface-active owing to their large specific surface areas^23^. Furthermore, these structures may be considered as periodic surface-nanostructured materials with a periodic arrangement of small ligaments and cavities. Such surface-nanostructured materials enable metallurgical bonding without the requirement of complex surface modifications using CMP or special cleaning processes. Furthermore, these characteristics allow for a low bonding temperature and pressure without using organic solvents or flux. Therefore, this approach using surface-nanostructured materials is expected to be a promising void-free and large-area-compatible solid-state bonding technology. Consequently, the elucidation of the solid-state bonding behavior of surface-nanostructured materials is expected to receive significant attention in the field of electronic device bonding.

The mechanisms of traditional solid-state bonding, commonly known as diffusion bonding, have been theoretically demonstrated^24,25^. The importance of interfacial plastic deformation, creep deformation, and surface/grain boundary diffusion for growing neck surfaces has been highlighted. Recently, molecular dynamics (MD) simulations have also demonstrated the diffusion bonding behavior on an atomistic scale. Chen et al.^26^ investigated the atomistic diffusion bonding mechanism of an Al-Cu joint at various temperatures, and surface deformation resulted in increased contact areas and the disappearance of initial gaps as the temperature increased, which was followed by the diffusion of atoms at a constant temperature. Moreover, they reported the influence of surface roughness on the bonding behavior. Li et al.^27^ also demonstrated the diffusion bonding mechanism in the Al-Cu joint and discussed the interdiffusion behavior across the ideally contacted Al-Cu interface. The main diffusion mechanism between Al and Cu was discussed from the perspective of activation energy. Xydou et al.^28^ investigated the interfacial void-closing behavior of Cu diffusion bonding and highlighted the importance of grain boundary diffusion for densification at the bonding interface. The MD simulation method can provide significant insights at the atomistic scale to elucidate the bonding behavior utilizing surface-nanostructured materials, whereas experimental approaches have limitations at this scale.

In this study, MD simulations are applied to elucidate the solid-state bonding behavior of surface-nanostructured materials, with a focus on the interfacial densification and diffusion behavior. A surface-nanostructured material was constructed as a simplified periodic ligament-cavity structure. Surface-nanostructured Cu and Au were chosen as bonding couples for the simulation because Cu-Au dissimilar bonding is commonly selected as a die-attached combination in power device applications. To focus on diffusion, which is one of the most important parameters affecting bonding behavior, simulations were conducted in the absence of the bonding pressure. Therefore, it is noteworthy that plastic deformation and creep deformation caused by the bonding pressure are beyond the scope of this study. First, the effect of the surface nanostructure, especially the specific surface area, on the bonding behavior was investigated. Subsequently, based on atomic displacement analysis, the effect of the specific surface area on the interfacial densification and diffusion behavior was evaluated. Lastly, the unique bonding behavior of surface-nanostructured materials is discussed in terms of the variations in atomic arrangements and diffusion coefficients.

## Methods

**Figure 1:**
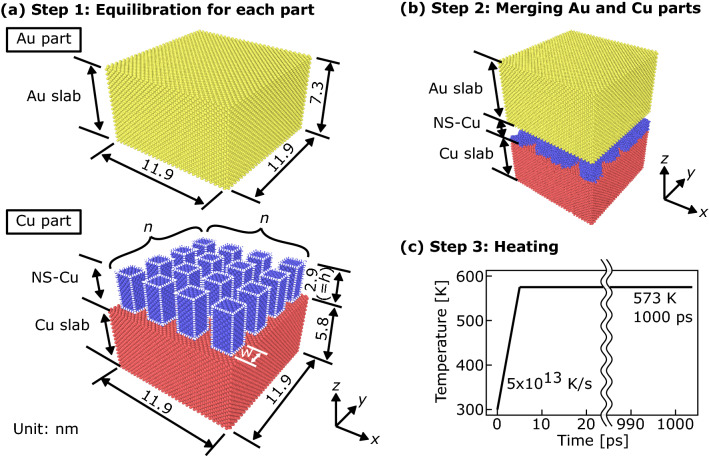
MD simulation setup of Cu and Au models; (**a**) step 1: equilibration of Au and Cu parts at 300 K for 20 ps; (**b**) step 2: merging of Au and Cu parts; (**c**) step 3: heating from 300 to 573 K at the heating rate of 5 × 10^13^ K/s, maintained for 1000 ps.

In this study, MD simulations were performed using the Large-scale Atomic/Molecular Massively Parallel Simulator (LAMMPS) software^29^. The well-established embedded atomic method (EAM) potential for Cu and Au reported by Zhou et al.^30^ was applied to describe atom interactions. The simulation procedure and model are illustrated in Fig. 1. The simulation steps applied were as follows: first, the Au and Cu parts were equilibrated separately; second, the Au and Cu parts were merged; third, bonding during the heating process was simulated.

Simulation models of the Au and Cu parts were prepared separately, as shown in Fig. 1a. The Au part was modelled as a monocrystalline Au slab. The Cu part consisted of a monocrystalline Cu slab and periodic nanostructured Cu (NS-Cu). NS-Cu was simplified as multiple monocrystalline cuboids with a uniform morphology arranged at regular intervals, to simulate a ligament-cavity geometry. The width (*w*) and number of cuboids (*n*^2^) were parametrically varied for the simulations. The height along the z-axis (*h*) was constant at 2.9 nm, which is equivalent to eight iterations of the Cu lattice. All the contact surfaces between the Cu slab, NS-Cu, Au slab, and boundary surfaces were (100) planes. The sizes of the Cu and Au slabs were determined by repeating the Cu lattice spacing (*a*_Cu_ = 0.36147 nm) in 32 × 32 × 16 and that of Au (*a*_Au_ = 0.40785 nm) in 28 × 28 × 18. The initial simulation box size along the x- and y-axes (*L*_x,0_ = *L*_y,0_) was 11.49 nm, which was assumed to reduce the size mismatch between the Cu and Au slabs along the x- and y-axes to approximately 0.64%.

The two initial shape parameters of NS-Cu–the specific surface area to volume ratio of NS-Cu (*S*_v_) and volume fraction (*V*_f_)–were defined. Specifically, *V*_f_ is the volume fraction of NS-Cu in the region of the simulation box occupied by the NS-Cu layer. The shape parameters were expressed as follows:

1 $${S}_{\mathrm{v}}=\frac{(4wh+{w}^{2}){n}^{2}}{{w}^{2}h{n}^{2}}=\frac{4}{w}+\frac{1}{h},$$

2 $${V}_{\mathrm{f}}={\left(\frac{n \cdot w}{{L}_{\mathrm{x},0}}\right)}^{2}.$$

The values of *S*_v_ and *V*_f_ used in this study are listed in Table 1. *S*_v_ was parametrically varied to 2.8, 2.2, and 1.6 nm^−1^ for simulations by changing *w* and *n*^2^, to elucidate the effect of nanostructure on bonding behavior. In this study, *V*_f_ was set as a constant of approximately 32 vol%. The number of atoms in these models was 133,008, 133,000, and 134,260.

**Table 1:** Shape parameters of NS-Cu in the simulations.

*h* [nm]	*w* [nm]	*n*^2^ [–]	*S*_v_ [nm^−1^]	*V*_f_ [vol%]
2.9	1.6	4 × 4	2.8	32
	2.2	3 × 3	2.2	32
	3.3	2 × 2	1.6	32

The initial velocities of the atoms were randomly assigned a Gaussian distribution for the set temperature. Newton's equation of motion was integrated with the Verlet algorithm, with a fixed time step of 1 fs. During the first step, the Au and Cu parts were equilibrated separately at 300 K for 20 ps, as shown in Fig. 1a. A periodic boundary condition was set for all boundary surfaces for Au equilibration. For the equilibration of the Cu part, periodic boundary conditions were implemented along the x- and y-axes; non-periodic and fixed boundary conditions were set along the z-axis. The boundary surface along the z-axis was set sufficiently far from the NS-Cu interface to model it as a surface. Equilibration was performed using the canonical (NVT) ensemble. In this study, the potential energy in the atomic slabs was confirmed to be well relaxed after equilibration. During the second step, the equilibrated Au and Cu parts were merged using the atomic-scale modeling software ATOMSK^31^, as shown in Fig. 1b.

During the third step, the temperature was rapidly increased from 300 to 573 K at a heating rate of 5 × 10^13^ K/s^26^. Subsequently, the temperature was kept constant at 573 K for 1000 ps, as shown in Fig. 1c. This simulation step was performed using the isobaric-isothermal (NPT) ensemble. Periodic boundary conditions were implemented in all the directions. The boundary surfaces at both edges along the z-axis were set sufficiently far from the NS-Cu/Au interface so that the interaction from these edge surfaces were negligible. During the simulations, an external pressure, equivalent to atmospheric pressure, was applied along the x-, y-, and z-axes. Many previous studies have demonstrated the necessity of applying a bonding pressure to achieve good interfacial bonding by enhancing plastic deformation at the contact surface. However, no bonding pressure other than atmospheric pressure was considered in this study, with the aim of focusing on diffusion-dominant behavior at the interface. The positions and velocities of the atoms were recorded every 1 ps. The atomic configurations were visualized using the Open Visualization Tool (OVITO)^32^.

## Results

### Morphology evolution of surface nanostructure

**Figure 2:**
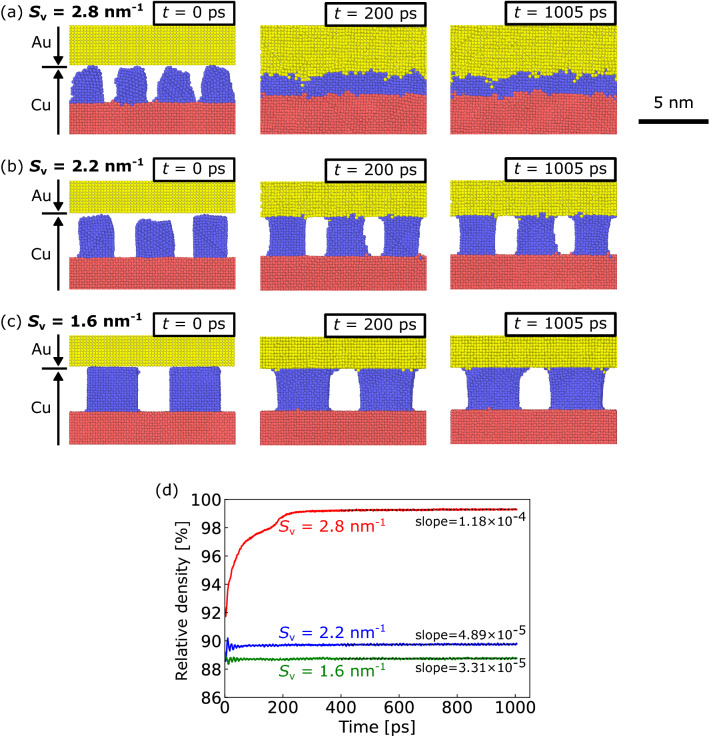
Evolution of the morphology of the surface nanostructure at the bonding interface. Cross-sectional configuration of the model with (**a**) *S*_v_ = 2.8, (**b**) 2.2, and (**c**) 1.6 nm^−1^ at time steps of 0, 200, and 1005 ps (only atoms near the interface are shown). Cu atoms in the Cu slab, NS-Cu, and Au atoms in the Au slab are depicted in red, blue, and yellow, respectively. (**d**) Relative density as a function of the simulation time for *S*_v_ = 2.8, 2.2, and 1.6 nm^−1^. Relative density evolution at *S*_v_ = 2.8 nm^−1^ was divided into an early stage (< 300 ps) and a later stage (> 300 ps). Linearly-fitted lines with slopes were inserted after 400 ps.

First, the evolution of the morphology of the surface nanostructure at the bonding interface was evaluated for various *S*_v_ values, as shown in Fig. 2. Figure 2a–c shows the cross-sectional configuration of the model with *S*_v_ = 2.8, 2.2, and 1.6 nm^−1^ at time steps of 0, 200, and 1005 ps, respectively. During these MD simulations, the densification behavior at the bonding interface was considered as an indicator of the bonding progress. Before heating, the NS-Cu cuboids were slightly deformed during the equilibration process, particularly at *S*_v_ = 2.8 and 2.2 nm^−1^. After heating, the model with *S*_v_ = 2.8 nm^−1^ exhibited rapid densification at the interface after 200 ps. Subsequently, no visible structural change was observed at 1005 ps, as shown in Fig. 2a. The Cu and Au slabs migrated closer to each other, with a significant displacement of Cu atoms in NS-Cu. Consequently, the initial cavities arranged within NS-Cu immediately disappeared. In contrast, in the models with *S*_v_ = 2.2 and 1.6 nm^−1^, as shown in Fig. 2b,c, respectively, the NS-Cu and Au slab contacted at the interface after 200 ps, accompanied by the minor diffusion of Au and Cu atoms along the surface. However, no significant densification was observed. The initial cavities arranged within the NS-Cu remained unchanged, even after the last simulation time step of 1005 ps. Although the significant displacement of atoms did not occur, atoms slightly migrated to the Cu and Au slab surfaces. All simulation results showed no apparent transfer of atoms across the interface through the interior of the slabs, which indicated that surface diffusion dominated the densification behavior. These results show the significant effect of *S*_v_ on densification at the interface.

Subsequently, the evolution of the relative density at the bonding interface was quantitatively investigated. The relative density (*d*_t_) at the simulation time step (*t*) of the simulation box was defined as follows:

3 $${d}_{t}=\frac{{d}_{\mathrm{apparent}, t}}{{d}_{\mathrm{ideal}}}\times 100 \left[\%\right],$$

where *d*_apparent,*t*_ is the apparent density of the simulation box at a time step of *t*, and *d*_ideal_ is the density of the ideal Cu/Au model with an ideal flat interface, no voids, and an equal number of atoms in each simulation. The values of *d*_apparent,*t*_ and *d*_ideal_ are expressed as the mass (*m*) divided by the simulation box volume at a time step of *t* (*V*_simbox,*t*_) and *m* divided by the simulation box volume of the ideal-Cu/Au model (*V*_ideal_), respectively. When *m* is constant, *d*_t_ is expressed as

4 $${d}_{t}=\frac{\frac{m}{{V}_{\mathrm{simbox},t}}}{\frac{m}{{V}_{\mathrm{ideal}}}}\times 100=\frac{{V}_{\mathrm{ideal}}}{{V}_{\mathrm{simbox},t}}\times 100 \left[\%\right].$$

Here, *V*_ideal_ was simulated at a temperature of 573 K for 1000 ps in advance. Figure 2d shows the relative density as a function of the simulation time for *S*_v_ = 2.8, 2.2, and 1.6 nm^−1^. The relative density evolution at *S*_v_ = 2.8 nm^−1^ was divided into early (< 300 ps) and later (> 300 ps) stages. During the early stages, the relative density rapidly increased with time, whereas during the later stages, the relative density slightly increased with time. The relative density at *S*_v_ = 2.2 and 1.6 nm^−1^ slightly increased without rapid densification compared with that observed at *S*_v_ = 2.8 nm^−1^. The rapid increase in the relative density at *S*_v_ = 2.8 nm^−1^ and slight variation at *S*_v_ = 2.2 and 1.6 nm^−1^ corresponded to the results shown in Fig. 2a–c. Moreover, the relative density was observed to increase with time, even at the later stage with *S*_v_ = 2.8 nm^−1^ as well as for *S*_v_ = 2.2 and 1.6 nm^−1^. A linear regression fit was used to calculate the slopes of the relative density curves, which were 1.18 × 10^–4^, 4.89 × 10^–5^, and 3.31 × 10^–5^%/ps for *S*_v_ = 2.8, 2.2, and 1.6 nm^−1^, respectively.

The slopes shown in Fig. 2d can be interpreted as the rate of densification. The result shows that densification gradually progressed in all the three cases (for *S*_v_ = 2.8, 2.2, and 1.6 nm^−1^). This behavior was observed even after rapid densification during the early stage at *S*_v_ = 2.8 nm^−1^. Furthermore, the slopes increased as the *S*_v_ values increase. Table 2 lists the predicted time to achieve a completely dense structure, *d*_t_ = 100%, which was calculated using the fitted regression line in Fig. 2d. The predicted times were 6.9, 210.2, and 341.1 ns for *S*_v_ = 2.8, 2.2, and 1.6 nm^−1^, respectively. This indicates that a larger surface area to volume ratio results in faster densification at a later stage. Therefore, the interfacial morphological evolution showed the importance of the surface nanostructure at the interface. This phenomenon is discussed in “Discussion” section.

**Table 2:** Predicted time to achieve a completely dense structure calculated using the fitted line from the relative density evolution.

*S*_v_ [nm^−1^]	Predicted time [ns]
2.8	6.9
2.2	210.2
1.6	341.1

### Diffusion between surface-nanostructured Cu and Au

**Figure 3:**
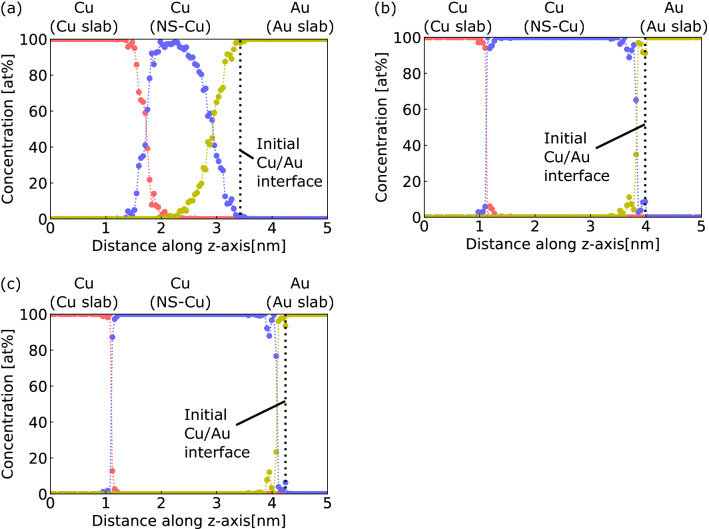
Concentration of Cu and Au atoms along the z-axis at a time step of 1005 ps and *S*_v_ = (**a**) 2.8, (**b**) 2.2, and (**c**) 1.6 nm^−1^. Red plots and line represent the Cu slab; blue plots and line represent NS-Cu; yellow plots and line represent the Au slab (only concentrations near the interface are shown).

To determine the diffusion thickness across the surface nanostructure, the concentration of Cu and Au atoms along the z-axis was obtained at a time step of 1005 ps for *S*_v_ = 2.8, 2.2, and 1.6 nm^−1^, as shown in Fig. 3 (only data near the interface are presented). The atomic concentrations along the z-axis were calculated as a ratio of the number of atoms filling each simulation region divided by 400 evenly-spaced planes parallel to the x–y plane. In this study, the concentrations were considered by neglecting the remaining cavities within the NS-Cu region. Figure 3a shows that both the Cu-slab (red) and NS-Cu (blue) curves intersect at *S*_v_ = 2.8 nm^−1^. This indicates that diffusion of the Cu slab into the NS-Cu cavities occurred. Similarly, the NS-Cu and Au-slab (yellow) curves intersected each other, which indicated that the diffusion of the Au slab into the NS-Cu cavities also occurred. In contrast, Fig. 3b,c show that there was no apparent diffusion layer between the Cu slab and NS-Cu or between the NS-Cu and Au slab at *S*_v_ = 2.2 and 1.6 nm^−1^. The diffusion thickness of the Au atoms was only a few atomic layers, even after the last time step in the simulation. These results suggest that diffusion was enhanced between NS-Cu and the Cu slab and NS-Cu and the Au slab only at *S*_v_ = 2.8 nm^−1^.

**Figure 4:**
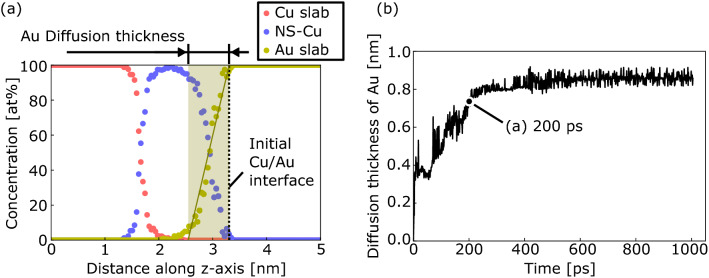
Evolution of the Au diffusion thickness at *S*_v_ = 2.8 nm^−1^. (**a**) Concentration of Cu and Au atoms along the z-axis at a time step of 200 ps, showing the Au diffusion thickness defined as the interfacial region with the concentration of Cu and Ag atoms over 5%, as shown in the yellow area with the fitted yellow line. (**b**) Au diffusion thickness versus time.

As described above, a clear interdiffusion layer was observed at *S*_v_ = 2.8 nm^−1^. Therefore, the evolution of the Au diffusion thickness at *S*_v_ = 2.8 nm^−1^ was investigated, as shown in Fig. 4. The concentration of Au along the z-axis (*C*_Au_) was used to determine the diffusion thickness of Au into the Cu region, as shown in Fig. 4a. First, a straight-line approximation of *C*_Au_ was obtained between *C*_Au_ = 5 and 95%. Subsequently, the distance along the z-axis between the two intersections of the fitted lines with *C*_Au_ = 0 and 100% was defined as the diffusion thickness of Au. Figure 4b shows the relationship between the Au diffusion thickness and time. The Au diffusion thickness significantly increased with time up to 300 ps, followed by a gradual increase. The Au diffusion thickness at 300 and 1005 ps were 0.80 and 0.86 nm, respectively. The evolution of the diffusion thickness can be divided into an early rapid diffusion stage and a later gradual diffusion stage, similar to the morphological evolution, as shown in Fig. 2d. This implies that both the morphological evolution and interdiffusion between NS-Cu and the Au slabs occurred via the significant displacement of NS-Cu atoms at the interface.

## Discussion

Simulations with various *S*_v_ values demonstrated the effect of surface nanostructures with a high surface area to volume ratio on rapid densification, accompanied by interdiffusion between Cu and Au atoms. Furthermore, it was suggested that this phenomenon involves significant displacement of atoms around the surfaces of NS-Cu. In contrast, in conventional solid-state bonding, interfacial densification is the dominant driving force for the interfacial diffusion of atoms, which is limited by temperature- and material-dependent characteristics^24,25^. However, surface-nanostructured materials may include shape-dependent characteristics as an additional driving force for rapid interfacial densification. Therefore, the novel bonding behavior of surface-nanostructured materials may be beneficial. Herein, we discuss the unique bonding behavior from the perspective of atom displacement at the bonding interface.

**Figure 5:**
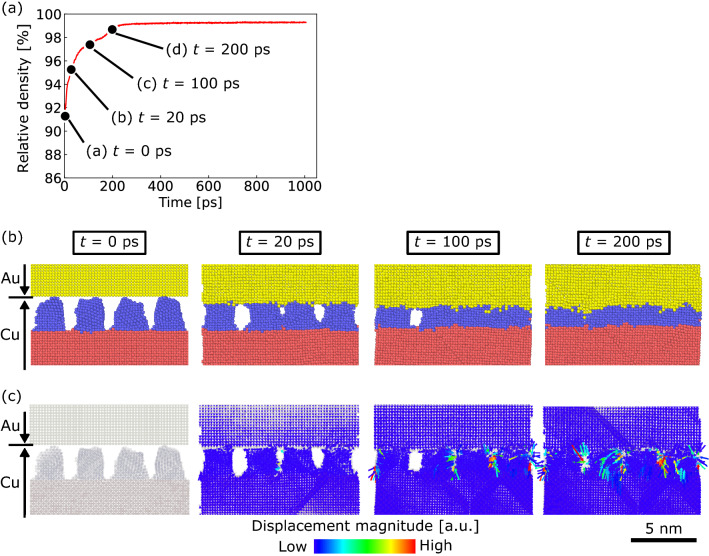
Atomic displacement behavior during the early rapid densification stage at *S*_v_ = 2.8 nm^−1^ at time steps of 0, 20, 100, and 200 ps; (**a**) corresponding relative density evolution, (**b**) cross-sectional snapshots, and (**c**) displacement vectors of atoms. Only atoms near the interface are shown for (**b**) and (**c**).

First, the atomic displacement behavior during the early rapid-densification stage at *S*_v_ = 2.8 nm^−1^ was further investigated, as shown in Fig. 5. Time steps of 0, 20, 100, and 200 ps were considered, as shown in Fig. 5a. Figure 5b shows cross-sectional snapshots taken during the simulation at these time steps (only atoms near the interface are shown). The NS-Cu surface contacted the Au slab surface within 20 ps. At this time step, the existing cavities between NS-Cu cuboids remained as gaps in the material. The smaller gaps disappeared by 100 ps, whereas the larger gaps did not. At 200 ps, all gaps in this cross-section disappeared entirely, which resulted in a highly densified interface. The displacement vectors of the atoms corresponding to the snapshots in Fig. 5b are shown in Fig. 5c. At 20 ps, small displacement vectors were observed in the smallest gap between the NS-Cu cuboids, as shown in the center of the figure. This indicates that a smaller gap would preferentially be filled. At 100 ps, displacement vectors were also observed in larger gaps, except for the largest gap in the cross section. Lastly, at 200 ps, displacement vectors appeared and filled the largest gap. These results indicate that displacement vectors were observed for the Cu slab, NS-Cu, and Au slab. This shows that interfacial densification behavior may have occurred because of the displacement of both Cu and Au atoms at the interface.

To quantitatively discuss the magnitude and direction of the atomic displacement, the mean square displacement (MSD) in the vicinity of NS-Cu was calculated as follows:

5 $$\mathrm{MSD}=\langle {r}^{2}(t)\rangle =\frac{1}{N}\sum_{i=1}^{N}\left({\left|{r}_{\mathrm{i}}\left(t\right)-{r}_{\mathrm{i}}(0)\right|}^{2}\right),$$

where *N* is the number of atoms, and *r*_i_(t) and *r*_i_(0) are the positions of atom *i* at times *t* and 0, respectively. Additionally, MSD_x_, which is the portion of the MSD along the x-axis, is expressed as follows:

6 $${\mathrm{MSD}}_{\mathrm{x}}=\frac{1}{N}\sum_{i=1}^{N}\left({\left|{r}_{\mathrm{x},\mathrm{i}}\left(t\right)-{r}_{\mathrm{x},\mathrm{i}}(0)\right|}^{2}\right),$$

where *r*_x,i_(t) and *r*_x,i_(0) are the x-positions of atom *i* at times *t* and 0, respectively. MSD_y_ and MSD_z_ were obtained in a similar manner. MSD is the sum of MSD_x_, MSD_y_, and MSD_z_. In this study, MSD_z_ was used as an indicator of the displacement that contributes to interfacial densification during bonding.

**Figure 6:**
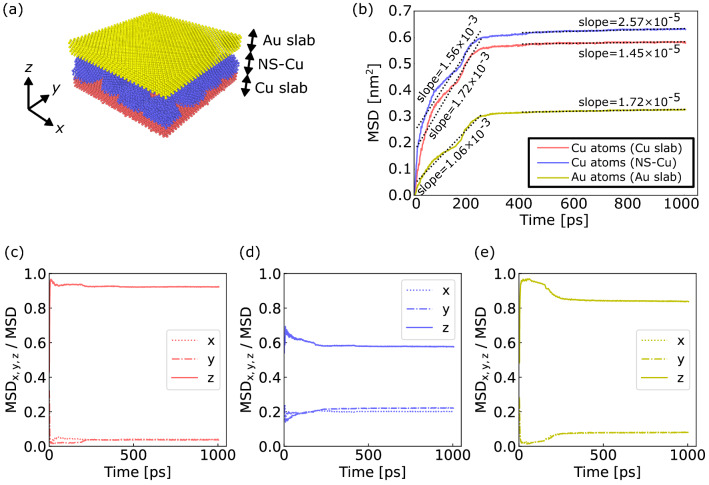
MSD evolution with time at *S*_v_ = 2.8 nm^−1^: (**a**) Number of atoms of the Cu slab, NS-Cu, and Au slab used in the MSD calculation was 11,027, 11,005, and 10,976, respectively. (**b**) The MSD evolution of each atom with linearly-fitted lines attached. MSDz to MSD ratio evolution of the atoms of the (**c**) Cu slab (red), (**d**) NS-Cu (blue), and (**e**) Au slab (yellow).

The MSD evolution of atoms of the Cu slab, NS-Cu, and Au slab in the vicinity of NS-Cu was evaluated at *S*_v_ = 2.8 nm^−1^, as shown in Fig. 6. The number of atoms used in the MSD calculation was selected to be approximately 11,000, as shown in Fig. 6a. Figure 6b shows the MSD curves of the atoms in the Cu slab, NS-Cu, and Au slab. Notably, the MSD of the Cu atoms was approximately twice as large as that of the Au atoms. This indicates that the contribution of the Cu displacement during the early rapid densification stage was larger than that of Au. Moreover, all curves had a shoulder at approximately 200 ps, which corresponds to the appearance of displacement vectors that filled the largest gap, as shown in Fig. 5. Furthermore, the MSD values significantly increased with time (< 300 ps), and subsequently, increased slightly (> 300 ps). This tendency was similar to that of the relative density (Fig. 2d) and diffusion thickness evolution (Fig. 4b).

Furthermore, the direction of the atomic displacement is discussed. Figure 6c–e shows the ratio of MSD_x_, MSD_y_, and MSD_z_ to the MSD of the Cu slab, NS-Cu, and Au slab at *S*_v_ = 2.8 nm^−1^. The values of MSD_x,y,z_/MSD range from 0 to 1, with a value close to 1 indicating that atomic displacement is highly unidirectional along the x-, y-, or z-axes, whereas a value of 0.33 shows isotropy. In the Cu slab, NS-Cu, and Au slab, the MSD_z_/MSD ratio was larger than the MSD_x_/MSD and MSD_y_/MSD ratios. Moreover, there was no significant difference between the MSD_x_/MSD and MSD_y_/MSD ratios. The MSD_z_/MSD values of the Cu and Au slabs were close to 1.0. The MSD_z_/MSD ratio was particularly large during the early rapid-densification stage (up to 300 ps). This suggests that the displacement of the atoms in the Cu and Au slabs in the vicinity of NS-Cu is highly unidirectional along the z-axis. Therefore, preferential atomic diffusion along the z-axis contributed to rapid interfacial densification.

Based on the previous results and discussions, each bonding behavior during the early and later densification stages is discussed in this section, in terms of the variation in the atomic arrangement and diffusion coefficient. Herein, the early stage refers to the period up to 300 ps, and the later stage refers to the period after 300 ps at *S*_v_ = 2.8 nm^−1^.

**Figure 7:**
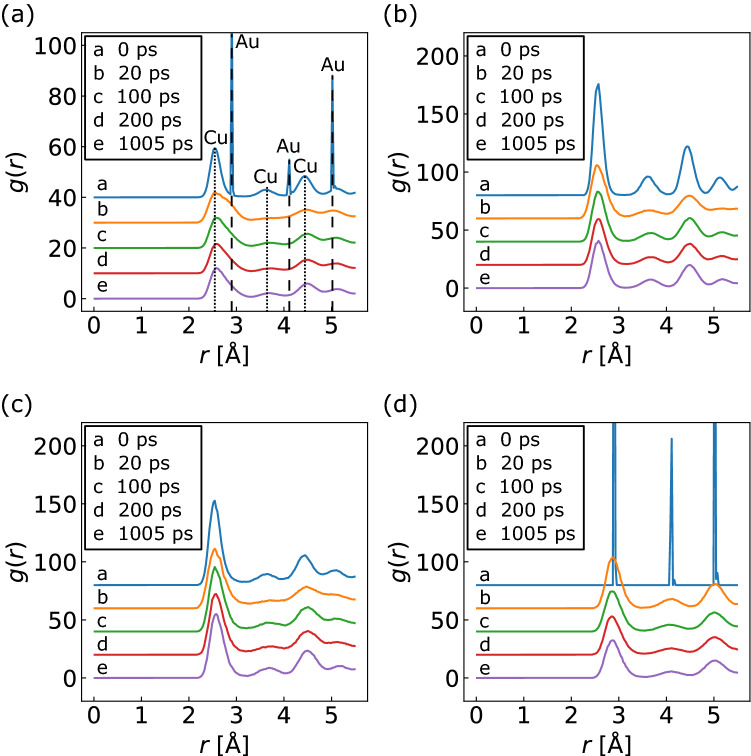
RDFs in the vicinity of NS-Cu at *S*_v_ = 2.8 nm^−1^ at time steps of 0, 20, 100, 200, and 1005 ps; (**a**) Cu and Au atoms in total, (**b**) only the Cu slab, (**c**) only NS-Cu, and (**d**) only the Au slab. Numbers of atoms of the Cu slab, NS-Cu, and Au slab used in the RDF calculation are the same as those in the MSD calculation, as shown in Fig. 6a.

The radial distribution function (RDF) *g*(*r*) was calculated to understand the atomic arrangement during bonding. The RDF is a well-known indicator that is presented as fluctuations in density around a given atom and describes the average number of atoms found at a given distance in all directions, which is expressed as follows^33^:

7 $$g\left(r\right)=\frac{V}{N}\frac{1}{4\pi {r}^{2}\Delta r}\frac{1}{N}\sum \limits_{i=1}^{N}{n}_{i}\left(r\right),$$

where *r* is the radial distance; *n*_*i*_ (*r*) is the coordination number of atom *i* separated by *r* within the Δ*r* interval; *V* is the volume of the system; *N* is the number of atoms. The RDF can provide insights into the atomic arrangement, with sharp peaks presenting a well-ordered crystal structure, whereas broad peaks present disordered structures, such as amorphous materials. The RDFs in the vicinity of NS-Cu at *S*_v_ = 2.8 nm^−1^ at the time steps of 0, 20, 100, 200, and 1005 ps are shown in Fig. 7. The atoms selected for the RDF calculations were the same as those for the MSD in Fig. 6a. Figure 7a shows the RDF of the Cu and Au atoms in total, where the number of atoms used for the calculation was 33,008. Figure 7b–d show the RDFs of the Cu slab, NS-Cu, and Au slab, respectively. The number of atoms used for these calculations was 11,027, 11,005, and 10,976, respectively. At a time step of 0 ps, sharp Au peaks and broad Cu peaks were detected, while the NS-Cu peaks were broader than the Cu slab peaks (as shown in Fig. 7b,c). Subsequently, the Au peaks broadened at a time step of 20 ps, as shown in Fig. 7d. The Cu slab peaks also became broader compared with that observed at 0 ps, as shown in Fig. 7b. Thereafter, the changes in these peaks were minor. This result indicates that the atomic arrangements were disordered during the very early densification stage upon contact between the Cu and Au atoms. This phenomenon may have induced early rapid densification between surface-nanostructured Cu and Au.

Subsequently, the bonding behavior in the early and later densification stages was investigated based on the diffusion coefficients. Using the Einstein’s diffusion law^34^, the diffusion coefficient is obtained as follows:

8 $$D=\underset{t\to \infty }{\mathrm{lim}}\frac{1}{2\widetilde{N}t}\langle {\left|r\left(t\right)-r(0)\right|}^{2}\rangle ,$$

where $\widetilde{N}$ is the dimensionality of the system, which is equivalent to the reciprocal of the MSD_z_/MSD ratio. Therefore, the diffusion coefficient was calculated using the slope of the MSD curve and MSD_z_/MSD ratio. For comparison with the results of this study, the surface self-diffusion coefficients (*D*) of Cu/Cu(100) and Au/Au(100) and the surface hetero-diffusion coefficients of Au/Cu(100) and Cu/Au(100) were calculated using the following Arrhenius equation:

9 $$D={D}_{0}\mathrm{exp}\left(-\frac{Q}{RT}\right),$$

where *D*_0_, *Q*, *R*, and *T* are the frequency factor, activation energy of surface diffusion, gas constant, and absolute temperature, respectively. The *Q* of Cu/Cu(100), Au/Au(100), Au/Cu(100), and Cu/Au(100) were 0.38, 0.64, 0.49, and 0.77 eV, respectively^35,36^. The *D*_0_ values of Cu/Cu(100) and Au/Au(100) are 1.20 × 10^–7^ and 0.80 × 10^–7^ m^2^/s, respectively^35^. Owing to limitations in the literature, the same *D*_0_ values for self-diffusion were used for Cu/Au(100) and Au/Cu(100).

**Figure 8:**
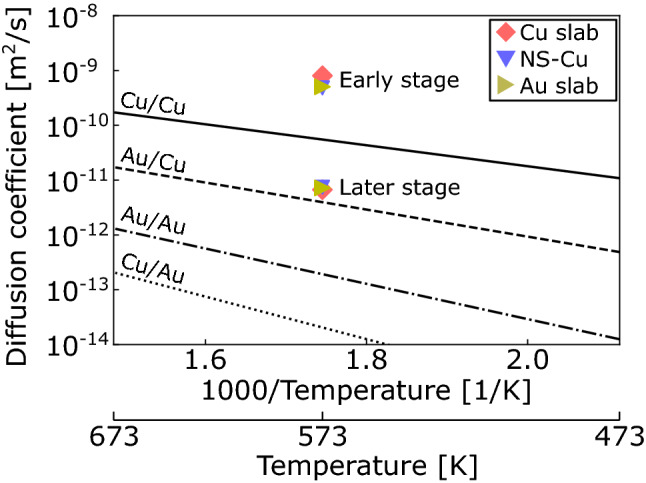
Diffusion coefficient of the Cu slab (red), NS-Cu (blue), and Au slab (yellow) at *S*_v_ = 2.8 nm^−1^ during the early stage (t = 10–250 ps) and later stage (t = 400–1005 ps). Surface self-diffusion coefficient of Cu/Cu(100) and Au/Au(100) and surface hetero-diffusion coefficient of Au/Cu(100) and Cu/Au(100) were inserted via the calculation using references^35,36^.

The diffusion coefficients of the Cu slab (red), NS-Cu (blue), and Au slab (yellow) at *S*_v_ = 2.8 nm^−1^ were obtained, as shown in Fig. 8. The diffusion coefficients were calculated from the results in the time step ranges of 10–250 ps and 400–1005 ps for the early and later densification stages, respectively. The difference between the Cu slab, NS-Cu, and Au slab at each stage was minimal. Notably, the diffusion coefficients for the early stage were higher than the highest surface diffusion coefficient of Cu/Cu observed at 573 K. This result may suggest a mechanism other than general surface diffusion mechanisms, such as simple jumps, long jumps, or exchanges. This high surface mobility can be rationalized by the fact that materials with a very high specific surface area, such as nanoparticles, exhibit significant coalescence behavior owing to their liquid-like surface layer^37^. The highly disordered atomic arrangement shown in Fig. 7 may confirm this suggestion.

In contrast, the diffusion coefficients at a later stage were intermediate between those of Cu/Cu and Au/Cu. This indicates that the well-known surface diffusion mechanisms dominate the bonding behavior at a later stage; this finding is within the scope of previously reported research on solid-state diffusion bonding^24,25^. These considerations suggest that a larger specific surface area enhances atom transport along the large surface, which resulted in faster densification, even during the later stage.

## Conclusion

This study investigated the solid-state bonding behavior between surface-nanostructured Cu and Au using MD simulations. The larger the specific surface area of the surface-nanostructured material, the faster the densification at the bonding interface. When the specific surface area was very large (*S*_v_ = 2.8 nm^−1^), the bonding process consisted of a rapid densification stage with significant interdiffusion, followed by a gradual densification stage. Atomic displacement analysis near the bonding interface indicated that the preferential diffusion of both Cu and Au atoms along the cavities inherent to the surface-nanostructured material contributed to the bonding behavior. Based on the relatively large diffusion coefficients observed during the rapid densification process compared with the typical surface diffusion coefficients, an atomic-transfer behavior unique to surface-nanostructured materials is proposed. The highly disordered atomic arrangement at the bonding interface played an essential role in the significant atomic diffusion. Additionally, the large surface area accelerated the bonding progress, even during the later gradual densification process, according to the typical surface diffusion mechanism. This study proves that surface-nanostructured materials exhibit good solid-state bonding performances and their use may serve as a suitable bonding strategy in a diverse range of electronic applications. Further investigations based on this method, which consider other important factors, such as the crystal orientation and bonding pressure, can provide valuable insights into the various fields of electronic packaging.

## Data Availability

All data generated or analyzed during this study are included in this published article, or are available from the corresponding author on reasonable request.
